# From Validation to Refinement: Optimising a Diagnostic Score for Atypical Lipomatous Tumours and Lipomas

**DOI:** 10.3390/diagnostics15243190

**Published:** 2025-12-14

**Authors:** Wolfram Weschenfelder, Katharina Lucia Koeglmeier, Friederike Weschenfelder, Till Rosenkranz, Christian Spiegel, Sebastian Walter

**Affiliations:** 1 Department of Trauma, Hand and Reconstructive Surgery and Orthopaedics, University Hospital Jena, 07747 Jena, Germanychristian.spiegel@med.uni-jena.de (C.S.); 2 Department of Obstetrics, University Hospital Jena, 07747 Jena, Germany; friederike.weschenfelder@med.uni-jena.de; 3 Department of Orthopaedic Surgery, University Hospital Cologne, 50937 Cologne, Germany; till.rosenkranz@uk-koeln.de (T.R.); sebastian.walter@ukbonn.de (S.W.); 4 Department of Orthopaedic Surgery and Traumatology, University Hospital Bonn, 53127 Bonn, Germany

**Keywords:** soft tissue tumour, lipoma, atypical lipomatous tumour, MRI, MDM2 amplification

## Abstract

**Background/Objectives**: Differentiating atypical lipomatous tumours (ALT) from lipomas remains challenging, as both share similar clinical and radiological features but require different forms of management. We previously proposed a clinical–radiological score integrating routine parameters to improve preoperative discrimination. This study aimed to externally validate the score in an independent cohort and refine it for enhanced robustness. **Methods**: We retrospectively analysed 119 patients with lipomatous tumours treated between 2022 and 2024 at an external university hospital. Diagnostic performance of the original models was assessed using receiver operating characteristic analysis. Data were then combined with the initial development cohort (n = 106) to recalibrate the models and define new cut-offs. **Results**: In the external validation cohort, predictive accuracy decreased compared to the derivation cohort, especially in extremity tumours assessed without contrast (AUC 0.830 vs. 0.942). Across four recalibrated models in the combined dataset (n = 225), diagnostic accuracy remained high (AUCs 0.918–0.954). Models combining clinical and imaging parameters consistently outperformed single-parameter approaches, with contrast enhancement providing the greatest incremental value. Accuracy was lower in trunk-localised tumours, highlighting the need for molecular confirmation in selected subgroups. **Conclusions**: The re-modelled score demonstrated robust diagnostic accuracy and practicality for routine use, offering a resource-efficient tool to support preoperative risk stratification. While molecular testing remains essential in high-risk cases, the refined score may reduce unnecessary testing and facilitate tailored diagnostic strategies. To support clinical adoption, the score is available as a web application that automatically selects the appropriate model and presents results in a colour-coded format.

## 1. Introduction

Adipocytic tumours constitute the most common subgroup of soft tissue neoplasms, representing roughly half of all benign lesions and a considerable proportion of soft tissue sarcomas. Benign soft-tissue tumours occur at an estimated annual incidence of approximately 3000 per 1,000,000 individuals, whereas soft-tissue sarcomas are far less common at around 50 per 1,000,000. Approximately 30% of benign soft-tissue tumours are lipomas. Clinically, both lipomas and soft-tissue sarcomas typically present as slowly growing, painless masses, making an initial clinical distinction difficult [1,2]. Within this spectrum, atypical lipomatous tumours (ALT) are defined as intermediate-grade according to the current WHO classification [3]. Differentiating ALT from simple lipomas is often problematic because both entities present with similar clinical and radiological appearances. The distinction, however, carries significant therapeutic implications: while lipomas may be observed or removed only for symptomatic relief, ALT require complete excision and routine follow-up owing to their tendency to recur and the possibility of dedifferentiation [4,5,6].

Accurate preoperative identification of ALT remains a diagnostic challenge. Standard evaluation relies on a combination of clinical data, imaging studies, and pathology. MRI is widely regarded as the most informative imaging modality [7], but in many cases the diagnosis can only be confirmed through detection of MDM2 amplification using fluorescence in situ hybridisation (FISH) [8,9]. Although highly reliable, this molecular test is expensive in time and money, labour-intensive, and not universally available. Various alternative strategies—including advanced MRI-based approaches, contrast-enhanced ultrasound, radiomics, and modified in situ hybridisation techniques—have been investigated, yet none has achieved broad clinical adoption to date [10,11,12,13,14,15,16].

In our earlier work, we addressed this diagnostic gap by proposing a clinical–radiological score designed to discriminate between ALT and lipomas. By integrating routine clinical parameters with MRI features, the score demonstrated excellent accuracy, especially in reliably ruling out ALT, thereby reducing the reliance on immediate molecular testing [17].

The present study was initially conceived as an external validation of this scoring system in an independent patient cohort. During this process, however, we identified opportunities to refine and optimise the model. We therefore re-modelled the score with the aim of improving its predictive performance and enhancing its applicability in routine clinical decision-making.

## 2. Materials and Methods

### 2.1. Study Population

For the external validation cohort, we retrospectively reviewed data from patients who underwent surgery for well-differentiated lipomatous tumours between January 2022 and December 2024 at a collaborating university hospital. Inclusion criteria were adult patients (≥18 years) with primary, well-differentiated lipomatous tumours and available adequate preoperative MRI performed for the assessment of a soft-tissue mass. Exclusion criteria were recurrent tumours and cases lacking sufficient preoperative imaging. None of these patients had been included in the development of the original model. In total, 119 eligible patients were identified. As the area under the curve (AUC) in this group showed notable deviations compared to the initial cohort, both cohorts were combined to re-optimise the four models. Including the 106 patients from the initial cohort, this resulted in a total of 225 patients for recalculating the models. The study was approved by the responsible ethics committees of the authors affiliated institutions (approval number 2025/3695-BO-D, 18 February 2025).

### 2.2. Data Collection and Prediction Models

Patient demographics, medical history, pathology reports, and imaging findings were extracted from medical records. MRI examinations were performed using both 1.5-Tesla and 3-Tesla scanners, employing dedicated soft-tissue tumour protocols tailored to the diagnostic question. All MRI scans were reassessed for homogeneity and for any degree of contrast enhancement, however minimal, by a musculoskeletal radiologist holding Level II certification in musculoskeletal radiology by the German Radiological Society and with long-standing experience in imaging of soft-tissue tumours at DKG-certified sarcoma centres. An orthopaedic oncology surgeon, fellowship-trained and certified by the German Orthopaedic Society (DGOOC), independently reviewed all cases. Diagnostic conclusions were reached collaboratively based on clinical data and imaging findings. Age and tumour size were transformed into binary variables according to the cut-off determined in the initial study. First, prediction models 1 to 4 from the previous study were tested on the external validation group [17]. Subsequently, the models were refined using the combined dataset.

Model 1 (“All combined”) includes seven preoperative variables: age (years), maximal diameter on MRI (centimetres), location in the lower limb, subfascial location, proximal location in the extremity, homogeneity on MRI, and contrast enhancement on MRI.

Model 2 (“without contrast”) incorporates six out of the seven variables that are obtainable when MRI is performed without contrast administration.

Model 3 (“Including trunk location”) uses six of these seven variables that apply when lesions of the soft tissues of the trunk are included.

Model 4 (“including trunk location without contrast”) is based on five of the seven variables accessible in non-contrast MRI, with the additional inclusion of soft tissue lesions located in the trunk.

### 2.3. Statistical Analysis

All statistical analyses were conducted using SPSS version 29.0 (IBM©, New York, NY, USA). All patients meeting the inclusion criteria and without exclusion criteria were analysed. Categorical variables were compared using the Chi^2^ test.

The statistical workflow followed three consecutive steps. First, the four previously published prediction models were applied to the external validation cohort. For each model, diagnostic accuracy was evaluated using the area under the curve (AUC) with 95% confidence intervals, as well as sensitivity and specificity based on the cut-offs defined in the original study [17].

Second, the same four original models were applied to the combined dataset of all 225 patients to assess their performance in a larger and more heterogeneous population. These results served as the reference for subsequent recalibration.

Third, binary logistic regression models were re-estimated in the full cohort to recalibrate the coefficients. New cut-offs were determined based on receiver operating characteristic analysis using both the Youden index and a threshold ensuring 100% sensitivity to avoid missing atypical lipomatous tumours. The diagnostic performance of the recalibrated models was then compared with both the original modelling results and the application of the initial models to the full cohort.

A *p*-value < 0.05 was considered statistically significant.

## 3. Results

### 3.1. Baseline Characteristics of External Validation Cohort

The external validation group comprised 53 females and 66 males. At the time of diagnosis, 89 patients were younger than 68 years, whereas 51 were older. A total of 101 patients had a lipoma, and 18 tumours were classified as atypical lipomatous tumours (all with confirmed MDM2 amplification). Additional baseline characteristics are provided in Table 1.

### 3.2. External Validation of Existing Prediction Models

Model 1 and Model 2 were applicable to 51 patients with tumours of the extremities (17 atypical lipomatous tumours and 34 lipomas). For Model 1, which included MRI contrast enhancement, the AUC was 0.913 (SD 0.040; 95% CI 0.835–0.991). For Model 2, where contrast enhancement was not considered, the AUC was 0.830 (SD 0.066; 95% CI 0.701–0.960).

**Figure 1:**
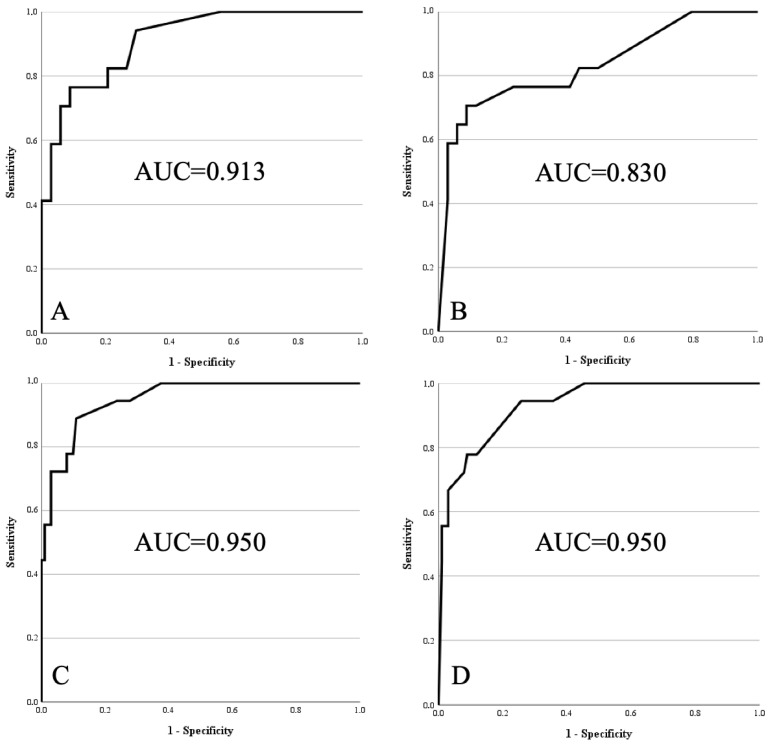
Receiver operating characteristics curves of models 1 to 4 within external validation cohort. (**A**) Model 1: age > 67 years, size > 15.5 cm, lower limb, deep, proximal, homogeneity on MRI, contrast enhancement on MRI. (**B**) Model 2: same as Model 1, without contrast enhancement. (**C**) Model 3: age > 67 years, size > 15.5 cm, lower limb, deep, homogeneity on MRI, contrast enhancement on MRI. (**D**) Model 4: same as Model 3, without contrast enhancement.

Model 3 and Model 4 were applicable to all 119 patients (18 atypical lipomatous tumours and 101 lipomas). Model 3, which included contrast enhancement, reached an AUC of 0.950 (SD 0.023; 95% CI 0.906–0.994). Model 4, which excluded contrast enhancement, yielded the same AUC (0.950; SD 0.023; 95% CI 0.906–0.994; see Figure 1).

For all models, diagnostic performance at the two predefined cut-offs (a: best Youden index in the initial cohort; b: 100% sensitivity in the initial cohort) is summarised in Table 2.

### 3.3. Baseline Characteristics of Combined Cohort

In the pooled dataset, 92 patients were female and 133 were male. At diagnosis, 159 individuals were below the age threshold of 68 years, while 66 were older. Overall, 170 cases were classified as lipomas and 55 as atypical lipomatous tumours; in all but one of the latter, MDM2 amplification was confirmed. Further baseline characteristics are summarised in Table 3.

### 3.4. Performance of the Original Models in the Combined Cohort

When applied to the full cohort of 225 patients, the original models achieved AUC values ranging from 0.904 (Model 2) to 0.940 (Model 3), indicating consistently high diagnostic performance. These results served as the reference for subsequent recalibration of the models.

### 3.5. Re-Optimised Models in the Combined Cohort

**Figure 2:**
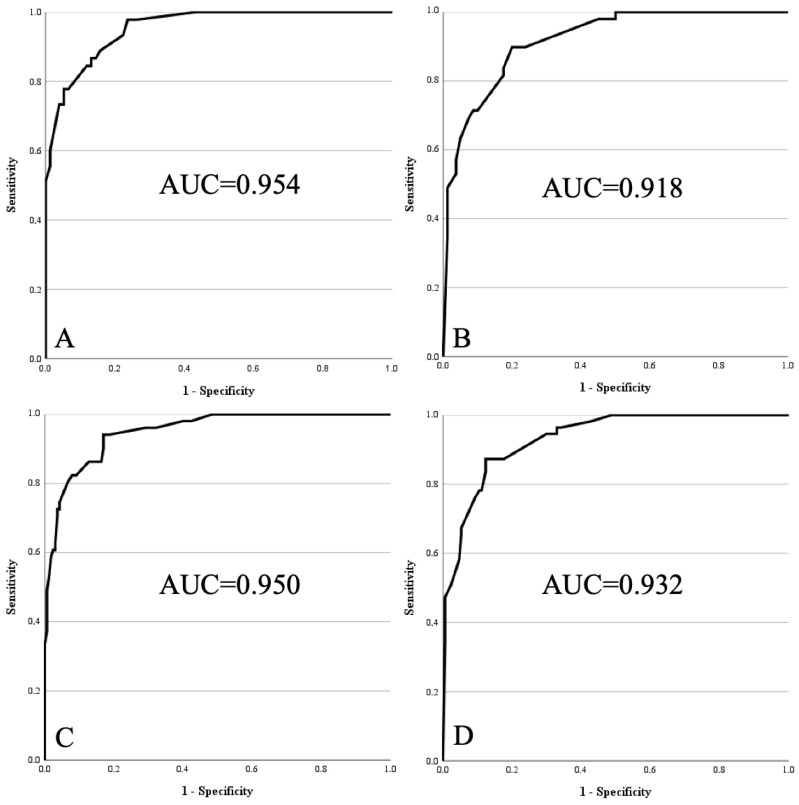
Receiver operating characteristics curves of re-optimised models 1 to 4 within pooled cohort. (**A**) Model 1: age > 67 years, size > 15.5 cm, lower limb, deep, proximal, homogeneity on MRI, contrast enhancement on MRI. (**B**) Model 2: same as Model 1, without contrast enhancement. (**C**) Model 3: age > 67 years, size > 15.5 cm, lower limb, deep, homogeneity on MRI, contrast enhancement on MRI. (**D**) Model 4: same as Model 3, without contrast enhancement.

After recalibration in the combined dataset, all four models demonstrated excellent diagnostic accuracy (Figure 2).

- Model 1 reached an AUC of 0.954 (95% CI 0.923–0.986) with good model fit (Chi^2^ = 95.436; df = 7; *p* < 0.001; Nagelkerke R^2^ = 0.744; Hosmer–Lemeshow *p* = 0.943).
- Model 2 achieved an AUC of 0.918 (95% CI 0.872–0.964; Chi^2^ = 81.709; df = 6; *p* < 0.001; Nagelkerke R^2^ = 0.638; Hosmer–Lemeshow *p* = 0.913).
- Model 3 yielded an AUC of 0.950 (95% CI 0.920–0.980; Chi^2^ = 134.572; df = 6; *p* < 0.001; Nagelkerke R^2^ = 0.697; Hosmer–Lemeshow *p* = 0.949).
- Model 4 resulted in an AUC of 0.932 (95% CI 0.899–0.966; Chi^2^ = 125.980; df = 5; *p* < 0.001; Nagelkerke R^2^ = 0.639; Hosmer–Lemeshow *p* = 0.919).

For each model, cut-offs were determined both by the Youden index and by maximising sensitivity to avoid overlooking atypical lipomatous tumours. Thresholds ranged from 0.1134 to 0.2595 (Youden) and from 0.0236 to 0.0755 (sensitivity-optimised). Predictive performance at these thresholds, including sensitivity, specificity, PPV, NPV, and the number of misclassified cases, is summarised in Table 4.

## 4. Discussion

This study aimed to externally validate our previously developed clinical–radiological scoring system for distinguishing ALT from lipomas. In the original population, the best-performing variant (Model 1) achieved an AUC of 0.952 [17]. External testing, however, revealed reduced predictive accuracy in certain settings. For example, in Model 2 (extremity tumours assessed without contrast), the AUC declined from 0.942 in the derivation cohort to 0.830 in the external group (Figure 1). After re-modelling, Model 2 reached an AUC of 0.918, representing the lowest but still robust performance among the revised models. This example illustrates the value of external validation and model adaptation. As a result, we not only confirmed the feasibility of the score in an independent cohort but also optimised it for greater robustness and clinical applicability.

In line with our initial findings, models that combined clinical and imaging parameters outperformed single-parameter approaches. Patient age, tumour size, and anatomical location again proved to be strong clinical discriminators, while MRI features such as lesion homogeneity and contrast enhancement remained decisive imaging markers [18,19,20]. These characteristics represent exactly those features that clinicians and radiologists routinely assess when evaluating lipomatous tumours, underscoring the practical relevance of the score in typical diagnostic workflows. The impact of contrast enhancement was particularly evident, as its exclusion led to a measurable drop in predictive accuracy, underlining the value of contrast-enhanced MRI in the diagnostic work-up of lipomatous tumours where not contraindicated [21].

Across all model versions, performance metrics remained high, with AUC values consistently above 0.918. Compared with the original variants, the re-optimised models achieved slightly more balanced calibration and goodness-of-fit, suggesting improved robustness in external cohorts. At the same time, our results indicate that predictive accuracy is reduced in certain subgroups, particularly trunk-localised tumours. In such cases, molecular pathology should remain the diagnostic standard. From a clinical perspective, this finding mirrors real-world diagnostic challenges: trunk lesions frequently reach substantial size, may display overlapping imaging features, and thus often require confirmatory MDM2 testing regardless of radiological appearance.

Artificial intelligence (AI)–based approaches have shown promising potential for differentiating ALT from lipomas, achieving very high accuracy in preliminary studies [22]. However, practical implementation is currently limited by the need for local software or secure image transfer and associated data privacy regulations. In daily practice, these constraints mean that AI tools are rarely available outside specialised centres, whereas our clinical–radiological score can be applied immediately using routine clinical data. In contrast, our web-based clinical–radiological score provides a globally accessible, easy-to-use tool for standardised evaluation without additional technical infrastructure.

In summary, our re-modelled clinical–radiological scoring system demonstrated robust diagnostic performance in an independent validation cohort. By integrating simple and routinely available parameters, the score provides a pragmatic alternative to more complex radiomics-based approaches and may help reduce unnecessary molecular testing in routine practice. Because it relies on information that is already part of standard diagnostic work-up, the score can be used at the first clinical presentation to support decision-making—particularly in settings without immediate access to molecular diagnostics or expert musculoskeletal radiology. These findings support its potential role as a resource-efficient tool for differentiating lipomas from ALT, while highlighting the need for further prospective validation.

### Strengths and Limitations

The main strength of this study is the external validation of our clinical–radiological score in an independent cohort, which allowed both confirmation and refinement of the original model. The re-optimised score remains practical for clinical use and compares favourably to radiomics-based approaches in terms of feasibility. Nevertheless, the overall sample size is limited, and predictive accuracy was lower in certain subgroups, such as trunk-localised tumours. Furthermore, intratumoral heterogeneity of MDM2 amplification means that molecular testing continues to play an indispensable role in selected high-risk cases [23]. Prospective multicentre validation will therefore be essential before widespread adoption.

## 5. Conclusions

Our study demonstrates that the originally developed clinical–radiological score for differentiating ALT from lipomas retains high diagnostic accuracy in an independent cohort. Re-modelling further improved its predictive performance while preserving simplicity and feasibility for routine use. Although molecular testing remains indispensable in selected high-risk cases, the refined score offers a practical tool to guide diagnostic decision-making and may help reduce unnecessary resource utilisation. To facilitate clinical adoption, we provide the score as a freely accessible web application that automatically selects the appropriate model and presents results in a clear, colour-coded format (https://drweschenfelder.de/alt-lipoma, accessed on 16 November 2025).

## Figures and Tables

**Table 1:** Baseline characteristics of external validation group (nominal data: number of cases and percentage of column, χ^2^-Test).

	Lipoma n = 101	Atypical Lipomatous Tumour (ALT) n = 18	*p*-Values
Age < 68 years	82 (81%)	7 (39%)	<0.01
Age ≥ 68 years	19 (19%)	11 (61%)	
Maximal diameter on MRI			<0.01
<15.5 cm	93 (92%)	6 (33%)	
≥15.5 cm	8 (8%)	12 (67%)	
Location			<0.01
- lower limb	34 (34%)	17 (94%)	
- other location	67 (66%)	1 (6%)	
Location in relation to fascia			<0.01
- superficial	45 (45%)	0 (0%)	
- deep	56 (55%)	18 (100%)	
Location within limb			0.05
- proximal	24 (71%)	16 (94%)	
- distal	10 (29%)	1 (6%)	
Homogeneity on T1 and STIR on MRI			<0.01
- yes	97 (96%)	7 (39%)	
- no	4 (4%)	11 (61%)	
Any contrast enhancement on MRI			<0.01
- yes	1 (1%)	16 (89%)	
- no	100 (99%)	2 (11%)	
Result of MDM2-amplification			<0.01
- positive	0 (0%)	18 (100%)	
- negative	101 (100%)	0 (0%)	

**Table 2:** Predictive accuracy of the four models in the external validation cohort (a = cut-off by Youden index in the initial cohort; b = cut-off with 100% sensitivity in the initial cohort; * number of cases per total cases). Values for missed ALTs and false positive lipomas are given as n/total cases.

Model	Sensitivity	Specificity	Positive Predictive Value	Negative Predictive Value	Missed Atypical lipomatous Tumours *	False Positive Lipomas *
Model 1a	0.824	0.735	0.609	0.893	3/17	9/34
Model 1b	0.941	0.706	0.615	0.960	1/17	10/34
Model 2a	0.824	0.529	0.467	0.857	3/17	16/34
Model 2b	1	0.206	0.386	1	0/17	27/34
Model 3a	0.778	0.911	0.609	0.958	4/18	9/101
Model 3b	1	0.545	0.281	1	0/18	46/101
Model 4a	0.778	0.901	0.583	0.958	4/18	10/101
Model 4b	0.944	0.644	0.321	0.985	1/18	36/101

Model 1: age > 67 years, size > 15.5 cm, lower limb, deep, proximal, homogeneity on MRI, contrast enhancement on MRI. Model 2: same as Model 1, without contrast enhancement. Model 3: age > 67 years, size > 15.5 cm, lower limb, deep, homogeneity on MRI, contrast enhancement on MRI. Model 4: same as Model 3, without contrast enhancement.

**Table 3:** Baseline characteristics of pooled cohort (nominal data: number of cases and percentage of column, χ^2^-Test).

	Lipoma n = 170	Atypical Lipomatous Tumour (ALT) n = 55	*p*-Values
Age < 68 years	137 (81%)	22 (40%)	<0.01
Age ≥ 68 years	33 (19%)	33 (60%)	
Maximal diameter on MRI			<0.01
<15.5 cm	150 (88%)	17 (31%)	
≥15.5 cm	20 (12%)	38 (69%)	
Location			<0.01
- lower limb	51 (30%)	47 (86%)	
- other location	119 (70%)	8 (14%)	
Location in relation to fascia			<0.01
- superficial	70 (41%)	2 (4%)	
- deep	100 (59%)	53 (96%)	
Location within limb			0.05
- proximal	58 (73%)	46 (94%)	
- distal	22 (27%)	3 (6%)	
Homogeneity on T1 and STIR on MRI			<0.01
- yes	142 (84%)	18 (33%)	
- no	28 (16%)	37 (67%)	
Any contrast enhancement on MRI			<0.01
- yes	33 (20%)	42 (82%)	
- no	132 (80%)	9 (18%)	
Result of MDM2-amplification			<0.01
- positive	0 (0%)	48 (98%)	
- negative	170 (100%)	1 (2%)	

**Table 4:** Predictive accuracy of the four models in the pooled cohort (a = cut-off by Youden index in pooled cohort; b = cut-off with 100% sensitivity in pooled cohort * number of cases per total cases). Values for missed ALTs and false positive lipomas are given as n/total cases.

Model	Sensitivity	Specificity	Positive Predictive Value	Negative Predictive Value	Missed Atypical Lipomatous Tumours *	False Positive Lipomas *
Model 1a N	0.978	0.750	0.698	0.983	1/45	19/76
Model 1b N	1	0.566	0.577	1	0/45	33/76
Model 2a N	0.898	0.800	0.733	0.928	5/49	16/80
Model 2b N	1	0.500	0.551	1	0/49	40/80
Model 3a N	0.941	0.830	0.632	0.979	3/51	28/165
Model 3b N	1	0.360	0.389	1	0/51	80/165
Model 4a N	0.873	0.876	0.696	0.955	7/55	21/170
Model 4b N	1	0.512	0.399	1	0/55	83/170

Model 1: age > 67 years, size > 15.5 cm, lower limb, deep, proximal, homogeneity on MRI, contrast enhancement on MRI. Model 2: same as Model 1, without contrast enhancement. Model 3: age > 67 years, size > 15.5 cm, lower limb, deep, homogeneity on MRI, contrast enhancement on MRI. Model 4: same as Model 3, without contrast enhancement.

## Data Availability

The data presented in this study are not publicly available but available on request from the corresponding author. The data are not publicly available due to privacy and ethical restrictions.

## Abbreviations

The following abbreviations are used in this manuscript:

AI	Artificial Intelligence
ALT	Atypical Lipomatous Tumour
AUC	Area under the curve
FISH	Fluorescence in situ Hybridisation
IQR	Inter Quartile Range
MDM2	Murine Double Minute 2
MRI	Magnetic Resonance Imaging
NPV	Negative Predictive Value
PPV	Positive Predictive Value
ROC	Receiver operating characteristics
WHO	World Health Organisation

## References

[1] Myhre-Jensen O. (1981). A consecutive 7-year series of 1331 benign soft tissue tumours. Clinicopathologic data. Comparison with sarcomas. Acta Orthop. Scand..

[2] Gatta G., Capocaccia R., Botta L., Mallone S., De Angelis R., Ardanaz E., Comber H., Dimitrova N., Leinonen M.K., Siesling S. (2017). Burden and centralised treatment in Europe of rare tumours: Results of RARECAREnet-a population-based study. Lancet Oncol..

[3] Le Nail L.R., Crenn V., Rosset P., Ropars M. (2022). Management of adipose tumors in the limbs. Orthop. Traumatol. Surg. Res..

[4] Gronchi A., Miah A.B., Dei Tos A.P., Abecassis N., Bajpai J., Bauer S., Biagini R., Bielack S., Blay J.Y., Bolle S. (2021). Soft tissue and visceral sarcomas: ESMO-EURACAN-GENTURIS Clinical Practice Guidelines for diagnosis, treatment and follow-up. Ann. Oncol..

[5] Yee E.J., Stewart C.L., Clay M.R., McCarter M.M. (2022). Lipoma and Its Doppelganger: The Atypical Lipomatous Tumor/Well-Differentiated Liposarcoma. Surg. Clin. N. Am..

[6] Gitto S., Interlenghi M., Cuocolo R., Salvatore C., Giannetta V., Badalyan J., Gallazzi E., Spinelli M.S., Gallazzi M., Serpi F. (2023). MRI radiomics-based machine learning for classification of deep-seated lipoma and atypical lipomatous tumor of the extremities. Radiol. Med..

[7] Alkaduhimi H., van der Linde J.A., Flipsen M., van Deurzen D.F.P., van den Bekerom M.P.J. (2016). A systematic and technical guide on how to reduce a shoulder dislocation. Turk. J. Emerg. Med..

[8] Clay M.R., Martinez A.P., Weiss S.W., Edgar M.A. (2016). MDM2 and CDK4 Immunohistochemistry: Should It Be Used in Problematic Differentiated Lipomatous Tumors?: A New Perspective. Am. J. Surg. Pathol..

[9] Thway K., Wang J., Swansbury J., Min T., Fisher C. (2015). Fluorescence In Situ Hybridization for MDM2 Amplification as a Routine Ancillary Diagnostic Tool for Suspected Well-Differentiated and Dedifferentiated Liposarcomas: Experience at a Tertiary Center. Sarcoma.

[10] Clay M.R., Martinez A.P., Weiss S.W., Edgar M.A. (2015). MDM2 Amplification in Problematic Lipomatous Tumors: Analysis of FISH Testing Criteria. Am. J. Surg. Pathol..

[11] Zhang H., Erickson-Johnson M., Wang X., Oliveira J.L., Nascimento A.G., Sim F.H., Wenger D.E., Zamolyi R.Q., Pannain V.L., Oliveira A.M. (2010). Molecular testing for lipomatous tumors: Critical analysis and test recommendations based on the analysis of 405 extremity-based tumors. Am. J. Surg. Pathol..

[12] Nagano S., Yokouchi M., Setoguchi T., Ishidou Y., Sasaki H., Shimada H., Komiya S. (2015). Differentiation of lipoma and atypical lipomatous tumor by a scoring system: Implication of increased vascularity on pathogenesis of liposarcoma. BMC Musculoskelet. Disord..

[13] Mick P., Seeberger A., Renkawitz T., Lehner B., Hariri M., Fischer C., Doll J. (2024). Contrast-enhanced ultrasound reveals perfusion differences between benign lipoma and semi-malignant atypical lipomatous tumors: A prospective clinical study. Ultraschall. Med..

[14] Farshid G., Otto S., Collis M., Napper S., Nicola M. (2023). Silver In Situ Hybridization for the Rapid Assessment of MDM2 Amplification in Soft Tissue and Bone Tumors. Validation Based on an Audit of 192 Consecutive Cases Evaluated by Silver In Situ Hybridization and Fluorescence In Situ Hybridization. Appl. Immunohistochem. Mol. Morphol..

[15] Mardekian S.K., Solomides C.C., Gong J.Z., Peiper S.C., Wang Z.X., Bajaj R. (2015). Comparison of Chromogenic In Situ Hybridization and Fluorescence In Situ Hybridization for the Evaluation of MDM2 Amplification in Adipocytic Tumors. J. Clin. Lab. Anal..

[16] Wang X.Q., Hsu A.T.Y.W., Goytain A., Ng T.L.T., Nielsen T.O. (2021). A Rapid and Cost-Effective Gene Expression Assay for the Diagnosis of Well-Differentiated and Dedifferentiated Liposarcomas. J. Mol. Diagn.

[17] Weschenfelder W., Koeglmeier K.L., Weschenfelder F., Spiegel C., Malouhi A., Gassler N., Hofmann G.O. (2025). Atypical Lipomatous Tumours vs. Lipomas: A Multimodal Diagnostic Approach. Diagnostics.

[18] Ballhause T.M., Korthaus A., Jahnke M., Frosch K.H., Yamamura J., Dust T., Schlickewei C.W., Priemel M.H. (2022). Lipomatous Tumors: A Comparison of MRI-Reported Diagnosis with Histological Diagnosis. Diagnostics.

[19] Brisson M., Kashima T., Delaney D., Tirabosco R., Clarke A., Cro S., Flanagan A.M., O’Donnell P. (2013). MRI characteristics of lipoma and atypical lipomatous tumor/well-differentiated liposarcoma: Retrospective comparison with histology and MDM2 gene amplification. Skeletal Radiol..

[20] Cheng Y., Ko A.T., Huang J.H., Lee B.C., Yang R.S., Liang C.W., Tai H.C., Cheng N.C. (2019). Developing a clinical scoring system to differentiate deep-seated atypical lipomatous tumor from lipoma of soft tissue. Asian J. Surg..

[21] Nardo L., Abdelhafez Y.G., Acquafredda F., Schirò S., Wong A.L., Sarohia D., Maroldi R., Darrow M.A., Guindani M., Lee S. (2020). Qualitative evaluation of MRI features of lipoma and atypical lipomatous tumor: Results from a multicenter study. Skeletal Radiol..

[22] Cay N., Mendi B.A.R., Batur H., Erdogan F. (2022). Discrimination of lipoma from atypical lipomatous tumor/well-differentiated liposarcoma using magnetic resonance imaging radiomics combined with machine learning. Jpn. J. Radiol..

[23] Kuhnen C., Mentzel T., Lehnhardt M., Homann H.H., Sciot R., Debiec-Rychter M. (2010). Lipoma and atypical lipomatous tumor within the same neoplasia: Evidence for a continuous transition. Pathologe.

